# Message framing and self-conscious emotions help to understand pro-environment consumer purchase intention: an ERP study

**DOI:** 10.1038/s41598-020-75343-8

**Published:** 2020-10-27

**Authors:** Muhammad Zubair, Sidra Iqbal, Sardar Muhammad Usman, Muhammad Awais, Ruining Wang, Xiaoyi Wang

**Affiliations:** 1 School of Management, Zhejiang University, Hangzhou, China; 2 Neuromanagement Lab, Zhejiang University, Hangzhou, China; 3 Department of Education, Shaheed Benazir Bhutto Women University Peshawar, Peshawar, Pakistan

**Keywords:** Attention, Decision

## Abstract

Message framing plays an important role in advertising strategies and has been studied from various perspectives in different behavioral researches. In this study Event Rated Potentials technique helped to examine the neural mechanism of message framing effect on self-conscious emotions of pride and guilt regarding consumer purchase intention in the context of green marketing. Behavioral results showed that participants ranked higher on positive framing compared to negative framing. ERP results declared that N1 component was elicited by positive framed message with emotion of pride and guilt, reflecting use of attentional resources to acquire potential benefits at first stage of processing emotional information. At the second stage P2 was higher for negative framing containing pride and guilt slogan, showing more attention towards processing emotional information about potential loss. At the third stage LPP component portray that both positive and negative framing is supported by guilt emotion when processing emotional information in decision making. Our results delivered strong evidence that how purchase intention can be mediated by message framing under the pride and guilt emotions in the context of green marketing.

## Introduction

As the uprising lift about environmental concerns turn out to be highly essential across business practices, companies have acknowledged the importance of environment safe marketing strategies and highly emphasized to adopt pro-environmental behavior and green advertising about their products and services^1^. In fact, green advertising conveys its message to the needs of pro-environmental consumers^2^. Environmental appeals have given promising attitudes and better purchase intentions^3^, whereas consumers emotional processing has an effective role in stimulating green purchase intentions^4–6^. In this regard an ideal convincing communication strategy often used to manipulate consumer behavior and attitude^5^ and to present objectively same information in terms of either the advantages gained or disadvantages suffered is called as message framing^7–10^.

The prospect theory declares that decision making can be influenced in response to objectively same information by whether it is presented as a potential gain or a potential loss^11^. An overview of the message framing portrays that positive and negative message framing has been practiced extensively to examine the potential gain and loss effects of different advertising strategies^7,12^. The companies concern in shaping a positively framed message is emphasizing the benefits to the environment after purchasing the green products ,while the companies concern in shaping a negatively framed message is to highlight potential destructive outcomes resulting from consumers’ willingness to purchase non green products^8,13,14^. Message framing holds a relevance to green marketing appeals which have selfless motivations and encouragement to acquire environment friendly behaviors^9^.

In the field of social marketing various motivational, individual and circumstantial factors are greatly considered for their influential role in environmental message framing^10,15^. When people assess their behavior in accordance with some personal and social standards it generates self-conscious emotions^16,17^. In this study we will focus on two self-conscious emotions, pride and guilt. Indeed, these two emotions seem like significant in the framework of pro-environmental behavior. Pride is a positive emotion that evokes pleasurable and self-confidant feelings worth^18,19^. Whereas guilt is a negative emotion that is experienced as apologetic, anxious and tense feeling^20,21^. Pride^16,17,22^ and guilt^23,24^ have some common characteristics. In fact, both of these emotions are evoked in result of responsible feelings for an act, and the evaluation of that act shows personal and social standards. Some limited studies report self-regulatory function of self-conscious emotions^25,26^, subsequently it helps in adapting people behavior to reflect their standards^27^.

Theory of Planned Behavior^28^, posit that intentions are the main predictors of behavior. Previous research reveals that pride and guilt are linked with pro-environmental behavior^29,30^. In their study^31^, found that pride and guilt reflect self-regulatory function and both of these emotions link the environment friendly intentions with personal norms, attitudes and social norms. A self-regulatory function was provided by both pride and guilt when studying consumer behavior in the context of environment friendly consumer choices^32^. Negative self-conscious emotions facilitate the relationship between attitudes and social norms on intentions, in both contexts of textbook piracy and condom use^17,33^. According to^34^, guilt join up with gain framed message is more influential to acquire environment friendly behavioral intention.

In the past few years research on emotions to examine framing effects has got an enhanced theoretical^35,36^ and empirical attention^37,38^. As the examination of framing effect with respect to self-conscious emotions appears in the shape of cognitive biasedness, but previous studies have examined framing effect from numerous behavioral perspectives. Various research studies have applied cognitive neuroscience methods to examine framing effects^39–41^. By using eye-tracking method^39^ found, larger number of active eye movements were induced by negative frames. While no moderating effect was found on purchase intention with eye-tracking method^41^. Functional magnetic resonance imaging (fMRI) technique revealed association of positive frames with reflexive brain regions, and negative frames with reflective brain regions^40^. By applying ERP method, it was found that positive and negative messages were processed differentially and provided dissimilar purchase intentions^42^.

In comparison with other neuroscientific methods, ERP technique provides high temporal resolution and excellent measure to track modulations of neural activity^43^. It’s valuable in a sense to differentiate early perceptual reactivity from more difficult and elaborate emotional processes^44^. The ERP components that occur up to 300 ms after the stimulus is presented to show a stage of attention, which possibly reflects early sensual encoding of stimulus that is emotionally significant^45,46^. In fact, this type of effect probably state more refined processing of emotional stimuli along with a rich emotional effect, that can be viewed in higher ERP components like P3 and Late Positive Potential^47^. In this study, the experimental stimuli would be positive and negative framed messages accompanied by emotional words of pride and guilt. The data would be analyzed based on the three ERP components, N1 (90–160 ms), P2 (200–300 ms) and LPP (500–700 ms) to elaborate the attentional and emotional mechanism. The N100 is a negative going wave that occurs approximately 100 ms post stimulus^48^. The N100 is modulated by attention^49^, emotion^50^, and congruence^51,52^.

Consumer decision making process in various marketing related spheres is illuminated by ERPs regarding emotions and consumer preferences^53,54^. ERP components that appear at the first 300 ms post stimulus are normally considered for sensory encoding of emotionally significant stimuli^46,55^. In this regard P2 is an early ERP component that is widely examined^56^. The P2 component is described as a positive going wave with a peak latency range of 150–250 ms, having sensitivity to emotional valence of stimuli^57,58^. Some of the studies have found that a higher P2 amplitude is recorded for negative than positive stimuli^58–60^.

LPP is a positive, long lasting wave with a time window of around 500–700 ms post stimulus^61–63^. In particular, LPP have a higher amplitude at centro-parietal sites for emotionally significant stimulus than for neutral stimulus^64^ such as images, faces^47,65^ and words^33,66^. This component may process task demands and reflects elaborate processing phase of attention, evaluation and emotional visual stimuli^67,68^. Some research studies have reported that LPP can differentiate emotional valence, resulting in different amplitudes for positive and negative words^68–70^.

A variety of the research studies have examined emotion’s role in framing effects from different perspectives^39,40^, but the literature lacks a clear synthesis of the current evidence addressing this issue. An neuroscientific method like ERP can thus help to provide more insights to construct a persuasive message design^42^. In fact, by using a neuroscientific method like ERP, this paper is first in its nature to uncover that effect of message framing on self-conscious emotions of pride and guilt regarding consumer purchase intentions in the context of green marketing. Explicitly, our main focus is to examine the brain regions where message framing affects self-conscious emotions of pride and guilt. We are looking that when the potential benefits and losses are portrayed by message framing, whether they are adopted by the feelings of pride or rejected by guilt? And how these positive and negative self-conscious emotions ultimately influence the consumer decision making and purchase intentions? Based on the above literature we have developed the hypothesis that the ERP components discussed above will explain the effect of different message framing on participants’ self-conscious emotions of pride and guilt regarding consumer environment safe purchase intentions.

## Method

### Participants

In this study twenty male Chinese post graduate students were taken to perform experiment. The participation in the experiment was requested to both the male and female students but the female students were unwilling to participate. All the students participated were from Zhejiang University, aged 20–27 years (M = 23.3 years, SD = 2.11 years). The participants were free from any neurological and psychiatric disease and had a normal or corrected to normal vision. As a result of abnormal EEG recordings, data of two participants was discarded. Eventually eighteen participants, aged 20–27 years (Mean = 23.23 years, SD = 2.19 years) were considered for this study. The protocol of this research was approved by the Ethics Committee of Neuromanagement Laboratory at Zhejiang University. All experiments were performed in accordance with relevant guidelines and regulations. Before experiment written informed consent were obtained from all the participants.

### Stimulus

Twenty pictures of the green products and four messages; positive and pride, positive and guilty, negative and pride and negative and guilty were included in the experiment. Each picture and message was presented on independent slide. The pictures used were of the daily life green products and all of them were taken from internet. The positive and pride message developed was “buying it makes you feeling pride”, positive and guilty message was “buying it makes you feeling not guilty”, negative and guilty message was “Ignoring it makes you feeling guilty” and negative and pride message was “Ignoring it makes you feeling not pride”. All the pictures and messages were developed by the authors and were evaluated and approved by five members of the Neuromanagement Lab. All the information was displayed in Chinese Language. In order to ensure a smooth flow and consistency in experiment background a uniform size of 840 by 640 pixels of product pictures was developed and a size of 1300 by 420 pixels was developed for the messages. This activity was done with the help of Photoshop software.

### Procedure

**Figure 1:**
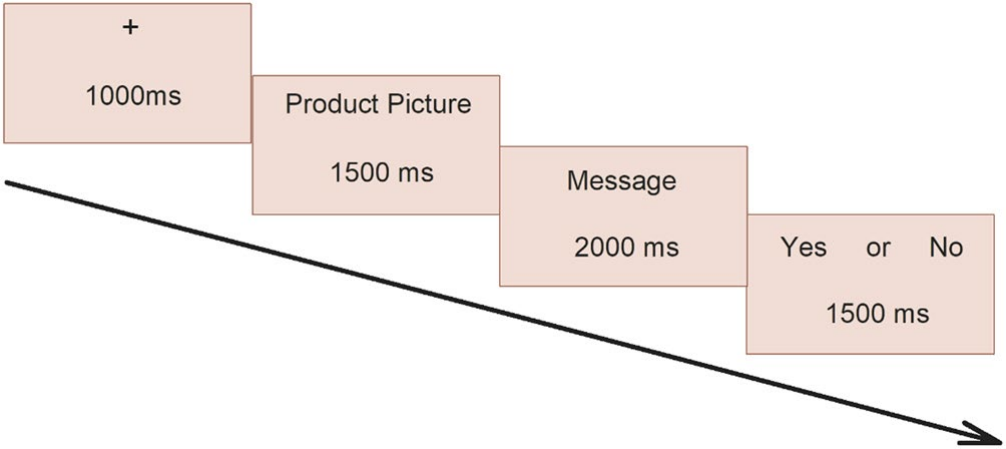
A single trial of the experiment procedure. Participants saw a green product picture first, then a message and then recorder their choice in the last.

All the participants received written instructions for conducting the experiment. The experiment was conducted in a dimly lit, electrically shielded room. The distance between each subject and computer screen was set as 100 cm. Participants used keyboard to record their choices. The experiment was designed in three unrepeated blocks. There were total 140 trials in the formal experiment and three practice trials were conducted before experiment to familiarize the participants with the experimental procedure. The experiment was run on E-prime 2.0 software package (Psychology Software tools, Pittsburgh, PA, USA). As shown in Fig. 1, the participants viewed in each trial a fixation point for 1000 ms then a product picture for 1500 ms followed by a message for 2000 ms and at the end a question about their choice of selection for 1500 ms. Based on the information provided in the message, the participants were asked are they willing to buy the product or not? The participants recorded their choices in the form of “Yes” or “No”, key 1 was used for Yes and key 3 was used for No.

### EEG recordings

The EEG data were recorded (band-pass filter of 0.05–70 Hz, sampling rate 500 Hz) with a NeuroScan SynAmps2 Amplifier (Scan 4.3.1, Neurosoft Labs, Inc., Virginia, USA). Data recording was done on 64 scalp sites with Ag/AgCl electrodes, according to the standard international 10–20 system. The electro-oculograms were recorded from the electrodes located near the outer canthus of each eye (Horizontal), above and below the left eye (Vertical). During the experiment electrode impedance remained below 5 kΩ. The left mastoid was used as a reference electrode. Offline, EEG data was referenced to the average of left and right mastoid. Digital filtering of EOG artifacts was done through a zero-phase shift (low pass at 30 Hz, 24 dB/octave). The EEG level for epochs was set between − 200 to 800 ms. Baseline correction was made 200 ms interval pre-stimulus onset. Artifacts were rejected beyond ± 80 μV.

The mean amplitude of the ERP components N1 (90–160 ms) was scored at F3/FZ/F4/FC3/FCZ/FC4/C3/CZ/C4, and P200 (200–300 ms) and LPP (500–700 ms) both were scored at C3/CZ/C4/CP3/CPZ/CP4/P3/PZ/P4 electrode sites^47,56,65,66,68^. Repeated measures ANOVA was conducted to obtain statistical results of the ERP^56,71^.

## Results

### Behavioral results

**Figure 2:**
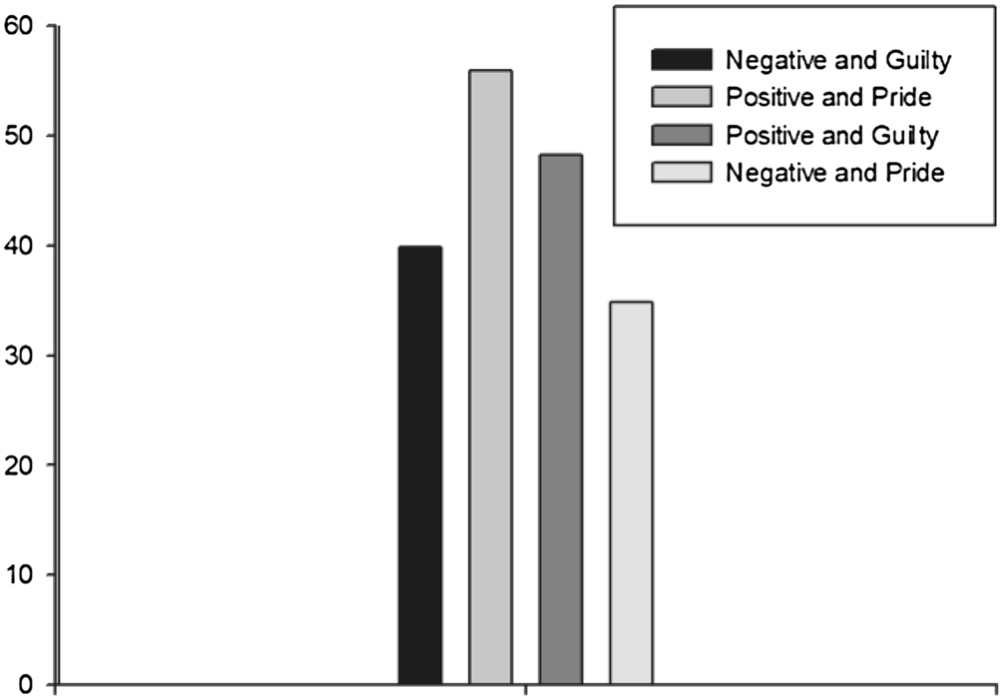
Purchase intentions of participants.

To compare the mean values of the three framing variables one-way ANOVA was used. According to the results of this test we found a significant effect for framing (F (1, 17) = 4.387, p = 0.05), but an insignificant effect for emotions (F (1, 17) = 0.095, p = 0.762). Figure 2 indicates the purchase intentions of the participants. The mean scores for positive message was (M = 52.07, SD = 6.62), and negative framing was (M = 37.30, SD = 7.82). The mean scores for emotion pride was (M = 45.35, SD = 6.22) and emotion guilt was (M = 44.02, SD = 7.13).

### ERP results

#### N1

Statistical analysis of repeated measures ANOVA showed a significant main effect for framing effect (F (1, 17) = 6.995, p = 0.017), whereas no significant effect for emotions (F (1, 17) = 0.596, p = 0.451) and electrodes (F (8, 136) = 1.590, p = 0.133) were found. Also, no significant interaction effects were found for framing, emotions and electrodes (F (8, 136) = 0.935, p = 0.490). Multiple comparisons gave the amplitude estimates for positive framing (Mean = − 1.333, SE = 0.354), negative framing (Mean = − 0.363, SE = 0.286), for emotion pride (Mean = − 1.043, SE = 0.374) and guilt (Mean = − 0.653, SE = 0.357). The mean unit of voltage value of the nine electrodes (F3/FZ/F4/FC3/FCZ/FC4/C3/CZ/C4) was − 0.8481 μV.

#### P2

Statistical analysis of repeated measures ANOVA showed a significant main effect for framing effect (F (1, 17) = 9.107, p = 0.008), and electrodes (F (8, 136) = 6.618, p = 0.001) were found, whereas no significant effect for emotions (F (1, 17) = 0.596, p = 0.188) was found. A significant interaction effects was found for framing and emotions (F (1, 17) = 9.791, p = 0.006). A simple effect analysis for this significant interaction effect was validated with the help of T-test. The results showed a significant effect for positive and pride message and negative and pride message (t = − 4.134, p = 0.001), while an insignificant effect was obtained for positive and guilty message and negative and guilty message (t = − 0.422, p = 0.678), this implies that positive and negative message under pride emotion has substantial effect which overcomes the insignificant effect of guilt emotion. Multiple comparisons gave the amplitude estimates for positive framing (Mean = 3.142, SE = 0.676), negative framing (Mean = 4.588, SE = 0.617), for emotion pride (Mean = 3.435, SE = 0.707) and guilt (Mean = 4.295, SE = 0.649). The mean unit of voltage value of the nine electrodes (C3/CZ/C4/CP3/CPZ/CP4/P3/PZ/P4) was 3.866 μV.

#### LPP

Statistical analysis of repeated measures ANOVA showed a significant main effect for framing effect (F (1, 17) = 5.508, p = 0.031), and electrodes (F (8, 136) = 4.807, p = 0.001) were found, whereas no significant effect for emotions (F (1, 17) = 1.574, p = 0.227) was found. Also, no significant interaction effects were found for framing, emotions and electrodes. Multiple comparisons gave the amplitude estimates for positive framing (Mean = 1.116, SE = 0.676), negative framing (Mean = 2.141, SE = 0.615), for emotion pride (Mean = 1.138, SE = 0.735) and guilt (Mean = 2.119, SE = 0.711). The mean unit of voltage value of the nine electrodes (C3/CZ/C4/CP3/CPZ/CP4/P3/PZ/P4) was 1.629 μV.

## Discussion

**Figure 3:**
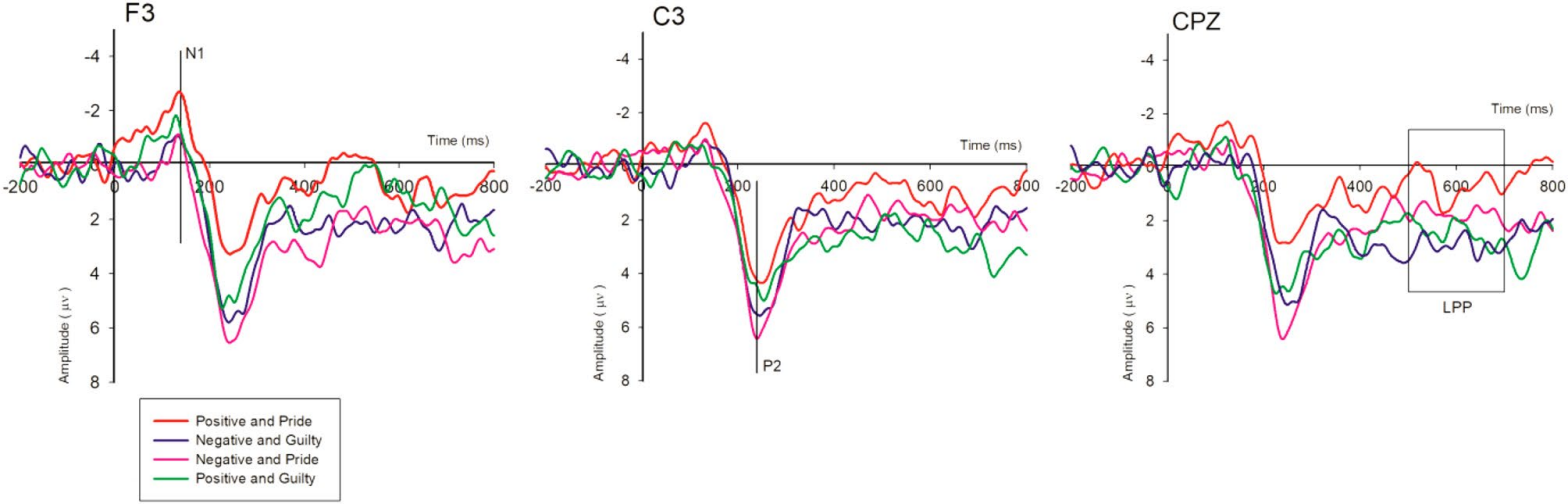
Grand average ERPs for all the four types of messages recorded at the indicated electrode sites.

The present study examined that how an emotional stimulus can evoke ERP components by using message framing in the context of Green Marketing. The results confirm the findings and supported both the prospect theory and the theory of planned behavior. We got evidence (see Fig. 3) that positive message framing including the pride and guilt emotions has greater impact on N1 component, while negative message framing with emotions of pride and guilt has more influenced the P2 component. Moreover, message framing both in positive and negative form along with the emotion of guilt has more influenced the LPP component.

N1 at frontal region is found to have reflection of both selective attentional processes^49,50^ and emotional processes^52,72^. Consistent with these studies our results also found N1 amplitude at the frontal regions. In a study by^73^, fearful expression evoked N1 component than happy and neutral faces at frontal brain site. Whereas our results elicited N1 component when the emotions of pride and guilt were influenced by positively framed message. A study by^50^, found that smaller N1 amplitude was produced by low frequency positive and negative words than neutral words. Whereas in our case we found that the positive message containing positive and negative emotional words of pride and guilt evoked N1 amplitude.

In their study^74^ they found that a better word recognition is reflected by a longer latency P2 component. P2 has the ability to show at first glance the affective content shown in stimuli^56,59^. The results of this study provided significant effect of negative framing messages designed along with emotional words of pride and guilt as compared to positive framing message. Negative framing message holds more attentional resources with larger P2 amplitude than positive framing message^42^. According to^73^, emotions of participants were activated by attractive and unattractive faces during logos recommendation experiment. On the other hand, our study declares that participant’s emotions were triggered by emotionally designed message framing. Although the P2 amplitude was enhanced in a social context, irrespective of the emotional significance of provided visual stimuli^75^. While our results in the context of green marketing reflected higher P2 amplitude for emotionally significant visual stimuli.

Our results found a significant effect of message framing on self-conscious emotions at the later cognitive stage of LPP. Some researchers have reported that stimuli based on emotional words evoked increased LPP amplitude^76–79^. Interestingly in the current study, our stimuli containing the emotional word guilt also evoked higher LPP amplitude for positive and negative framing. According to some previous studies the processing of positive and negative emotional information of stimuli is amplified by the expectation of socially important events^80–82^. Also Larger LPP amplitude was evoked by providing stimuli based on emotional words^83,84^. Whereas the findings of our study reveal that stimuli containing emotional word guilt, have provided enhanced LPP amplitude in the context of environment safety. The research studies worked on interactions of task and emotion have provided fronto-central LPP effects^46,85^. In the current study, centro-pareital effects are found for guilt emotion under the positive and negative framed messages. And the LPP amplitude produced by positive and negative words as compared to neutral words shows that the contents of the message are deeply processed^78^. Likewise, the emotional content guilt in our messages is deeply processed by the participants under the positive and negative combinations of message framing.

The results of this study found significant effect for various messages framed with emotional words. The information of the messages was processed at different regions of the brain and reflected a non-similar purchase intention. Although there is prominent evidence on framing effect in several studies but our main focus was to investigate underlying neural mechanism for the message framing effect on positive and negative self-conscious emotions regarding consumer purchase intention in the context of green marketing. For this reason, we used ERP method and tried to explain why self-conscious emotions affected by message framing can work under the umbrella of green marketing. In our ERP study we found some components related to message framing in green marketing appeals. We found the components in three stages. In the first stage N1, in the second stage P2, while LPP component was found in the third stage. The findings revealed consistency of the ERP components with the hypothesis that there is a substantial effect of different message framing on participants’ self-conscious emotions of pride and guilt regarding consumer environment safe purchase intentions. This was due to the reason that these messages contain a slogan and direction to indulge in environment safe or unsafe action. The results declared that positive and negative messages framed with emotions of pride and guilt functions in the context of green marketing. It allows the insight that these emotions enabled the participants to acquire a purchase intention according to their social norms towards environment safety.

Talking about the first stage at a very early attentional level N1, we found that participants adopted the positive framing along with pride and guilt. It shows that just confronting the message inclined them to acquire the potential benefits stated in the positive framing. Also, at this level the emotional effect indicates the pursuit of positive emotion pride and avoidance of negative emotion guilt in the context of pro-environmental behavior. The underlying neural mechanism at a very early stage reflects that self-conscious emotions guide positive framing towards environment safe purchase intention. Whereas on the second stage at P2, participants ranked the negative framing influenced greater than positive framing. At this level even both the emotions pride and guilt played their significant role. The participants have processed the information given in the messages, and tend to show environment safe behavior with their purchase intention. Same like the intention at early stage, here they have promoted both the pursuit of pride and avoidance of guilt.

In the last stage at LPP, the participants adopted both the positive and negative framing along with self-conscious emotion of guilt. Interestingly this indicates that the participants have processed the stimuli information and they have preferred avoidance of guilt in both shapes of positive and negative framing. It shows in their decision-making process emotion of guilt play important role than pride.

There are some practical implications of this study. In this case, first marketers should try to construct their advertising claims with the specific emotional words that drive the customer purchase intentions. Positively framed messages induced with self-conscious emotional words will attract attention of the customers at a very first glance. Negatively framed messages would also help in the case when customers go deeper to understand the message slogan. Second, by using self-conscious emotional words in advertising would help marketers to secure early attention plus the encouragement to acquire pro-environmental behavior.

Third our research sheds light on the value and role of neuroscience method to study consumer immediate brain response. Prior research has done behavioral studies to examine message framing and did not consider framing effects in green marketing context. Even none of the studies have used the emotional words to identify the framing effect. However, our results indicate that emotionally framed messages work in Chinese culture. Eventually it is clearly reflected that ERP is a fertile method to study customer motivational engagement with emotional significant marketing stimuli.

The current findings support the method of laboratory experiments with neuroscientific method to examine marketing promotional activities. Investigations of environment friendly advertising messages through Neuromarketing could improve understanding of scholars about underlying neural mechanisms of brain. Furthermore, these insights could enable marketers with improved predictions about consumer behavior in Chinese markets.

Further research could examine, if the advertisement and customer promotional activities have more things like packaging, pricing then it will change the customer thinking and judgment. Based on the ERP results framing effect along with pride and guilt emotions can work at the attentional and emotional stage. Including some other self-conscious emotional words may change the customer preference to deeply involve in environment safe purchase intentions. The participants in this study were all the male students, in the future studies to examine the message framing effect could be investigated both from male and female subjects of a broader age group. We made stimuli containing positive and negative messages along with emotional words of pride and guilt in this study. Further work by examining the verbs, nouns and other types of information structuring used in green marketing context could be analyzed in the future studies.

## Conclusion

In the current study we used message framing made-up with emotional words of pride and guilt in the green marketing context. In our real life it is very special pertaining to environmental safety and protection and is related to emotions. This study was performed to examine the attentional and emotional processing of message framing and its effect on self-conscious emotions resulting in ERP components modulation due to these messages. We found important contribution to ERP components like N1, P2, and LPP. Previous literature of behavioral studies showed these components were attracted by attention and related with early stage emotions. Whereas our findings from neural perspective indicate the message framing effect based on self-conscious emotions of pride and guilt.

## Data Availability

The datasets generated and/or analyzed during the current study are available from the corresponding author on reasonable request.
